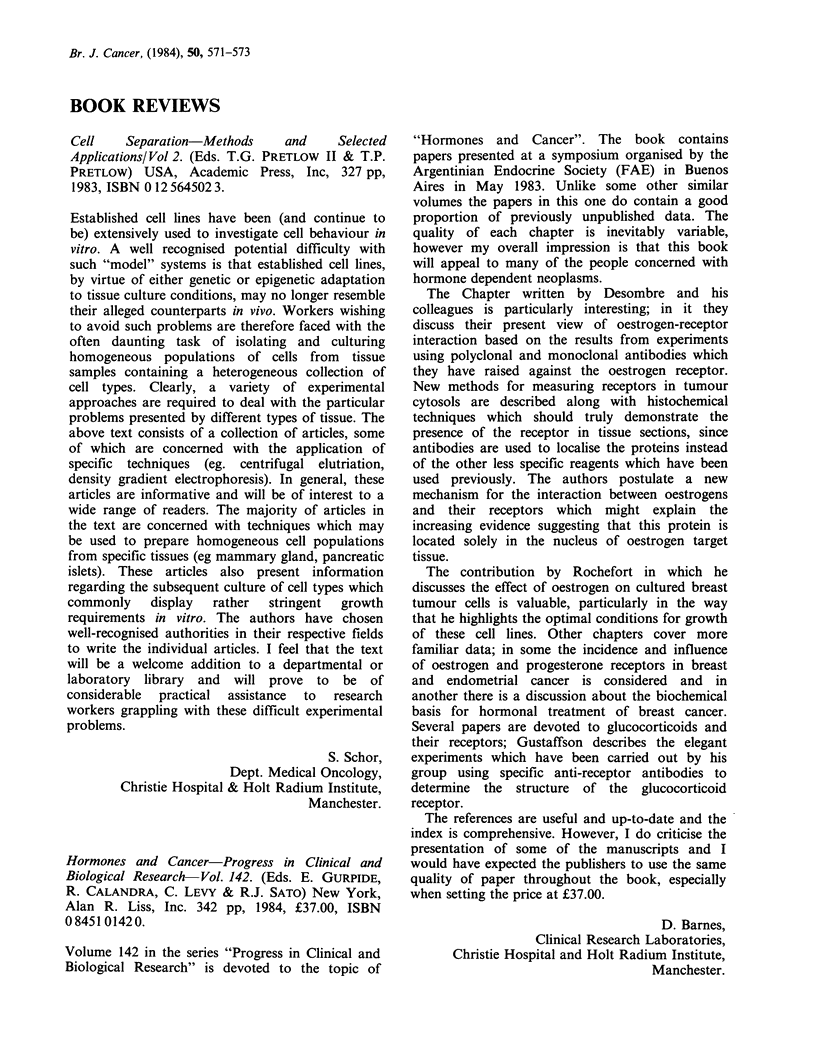# Hormones and Cancer—Progress in Clinical and Biological Research—Vol. 142

**Published:** 1984-10

**Authors:** D. Barnes


Manchester.

*Hormones and Cancer—Progress in Clinical and Biological Research—Vol. 142.* (Eds. E. GURPIDE, R. CALANDRA, C. LEVY & R.J. SATO) New York, Alan R. Liss, Inc. 342 pp, 1984, £37.00, ISBN 0 8451 0142 0.

Volume 142 in the series "Progress in Clinical and Biological Research" is devoted to the topic of "Hormones and Cancer". The book contains papers presented at a symposium organised by the Argentinian Endocrine Society (FAE) in Buenos Aires in May 1983. Unlike some other similar volumes the papers in this one do contain a good proportion of previously unpublished data. The quality of each chapter is inevitably variable, however my overall impression is that this book will appeal to many of the people concerned with hormone dependent neoplasms.

The Chapter written by Desombre and his colleagues is particularly interesting; in it they discuss their present view of oestrogen-receptor interaction based on the results from experiments using polyclonal and monoclonal antibodies which they have raised against the oestrogen receptor. New methods for measuring receptors in tumour cytosols are described along with histochemical techniques which should truly demonstrate the presence of the receptor in tissue sections, since antibodies are used to localise the proteins instead of the other less specific reagents which have been used previously. The authors postulate a new mechanism for the interaction between oestrogens and their receptors which might explain the increasing evidence suggesting that this protein is located solely in the nucleus of oestrogen target tissue.

The contribution by Rochefort in which he discusses the effect of oestrogen on cultured breast tumour cells is valuable, particularly in the way that he highlights the optimal conditions for growth of these cell lines. Other chapters cover more familiar data; in some the incidence and influence of oestrogen and progesterone receptors in breast and endometrial cancer is considered and in another there is a discussion about the biochemical basis for hormonal treatment of breast cancer. Several papers are devoted to glucocorticoids and their receptors; Gustaffson describes the elegant experiments which have been carried out by his group using specific anti-receptor antibodies to determine the structure of the glucocorticoid receptor.

The references are useful and up-to-date and the index is comprehensive. However, I do criticise the presentation of some of the manuscripts and I would have expected the publishers to use the same quality of paper throughout the book, especially when setting the price at £37.00.

D. Barnes,
Clinical Research Laboratories,
Christie Hospital and Holt Radium Institute,
Manchester.